# Tcf7l1 directly regulates cardiomyocyte differentiation in embryonic stem cells

**DOI:** 10.1186/s13287-018-1015-x

**Published:** 2018-10-11

**Authors:** Rui Liang, Yu Liu

**Affiliations:** Department of Biology and Biochemistry, University of Houston, Houston, TX 77004 USA

**Keywords:** Wnt, β-Catenin, T-cell factor/lymphoid enhancer factor, Tcf3, Cardiac myocytes

## Abstract

The T-cell factor/lymphoid enhancer factor (TCF/LEF) family protein Tcf7l1 is highly abundant in embryonic stem cells (ESCs), regulating pluripotency and preparing epiblasts for further differentiation. Defects in the cardiovascular system in *Tcf7l1*-null mouse were considered secondary to mesoderm malformation. Here, we used temporally controlled Tcf7l1 expression in *Tcf7l1*-null ESCs to address whether Tcf7l1 directly contributes to cardiac forward programming. Tcf7l1 knockout during differentiation impaired cardiomyocyte formation but did not affect mesoderm formation. *Tcf7l1*-null ESCs showed delay in mesoderm formation, but once completed, ectopic Tcf7l1 augmented cardiomyocyte differentiation. Further, Tcf7l1-VP16 and Tcf7l1dN showed procardiac activity whereas Tcf7l1-En was ineffective. Our results support that Tcf7l1 contributes to cardiac lineage development as a β-catenin-independent transactivator of cardiac genes.

**Electronic supplementary material:**

The online version of this article (10.1186/s13287-018-1015-x) contains supplementary material, which is available to authorized users.

## Introduction

The Wnt/ β-catenin signaling pathway is critical in stem cell pluripotency, differentiation and homeostasis [1, 2]. In the absence of WNT ligand, β-catenin is phosphorylated by a destruction complex composed of adenomatous polyposis coli (APC), glycogen synthase kinase 3 (GSK3), and kinases casein kinase 1 (CK1) [3]. Phosphorylated β-catenin is ubiquitinated and degraded by proteasomes. WNT ligand binding disaggregates the destruction complex, and in turn stabilizes β-catenin. Next, β-catenin is translocated into the nucleus where it binds T-cell factor/lymphoid enhancer factor (TCF/LEF) family proteins to transactivate downstream genes [4]. The Wnt/ β-catenin pathway plays a biphasic role in cardiogenesis: an initial activation phase in which Wnt/β-catenin promotes mesoderm formation, followed by an inhibitory phase in which the pathway is shut off to allow cardiac gene expression.

There are four TCF/LEF family proteins in mammals: TCF7, LEF1, TCF7l1, and TCF7l2 [5]. They bind the consensus DNA element 5′-(A/T)(A/T)CAAAG-3′ [3, 4]. Their interactions with both β-catenin and the transregulatory element are necessary for activating target genes in response to WNT signaling [6–8]. In mouse genetic studies, only Tcf7l1 deletion led to severe embryonic defects and lethality. The defects are related to delayed mesoderm specification, axis mesoderm duplication, and impaired lateral mesoderm formation. Some severely affected embryos display enlarged cardiac sacs, missing hearts, and multiple large blood vessels [9]. In embryonic stem cells, Tcf7l1 negatively modulates the expression of pluripotent genes, and prepares the epiblast for transition to lineage specification [10–12]. It has been reported that Tcf7l1 can function independently of β-catenin during gastrulation and hypothalamopituitary (HP) axis formation [3, 4, 13]. Because of defective mesoderm formation, whether Tcf7l1 intrinsically contributes to cardiac development has not been determined in *Tcf7l1*^−/−^ embryos.

Herein, based on a *Tcf7l1*^−/−^ background, we conducted temporally controlled Tcf7l1 rescuing experiments, and demonstrate that Tcf7l1 acts as an activator-like transcription factor and regulates cardiac lineage development independent of β-catenin.

## Materials and methods

**Cell culture**

*Tcf7l1*^+/+^ and *Tcf7l1*^−/−^ ESC lines were provided by Dr Bradley J. Merrill (University of Illinois at Chicago, USA). ESCs were propagated in 0.1% gelatin-coated dishes and cultured with feeder-free ESC medium (DMEM (Gibco) supplemented with 15% FBS (Atlanta Biologicals), 100 U/ml penicillin G, 100 μg/ml streptomycin sulfate, 2 mM l-glutamine, 0.1 mM β-mercaptoethanol, 1× 103 U/ml murine leukemia inhibitory factor (LIF; Global Stem)). The medium was changed daily. To induce EB formation and differentiation, the ESCs were grown as 20 μl hanging droplets (2 × 10^4^ cells/ml) in SFDM without LIF [1]. EBs were collected as indicated and the medium was replaced every 2 days. 293FT cells were cultured in DMEM (Gibco) supplemented with 20% FBS (Atlanta Biologicals), 100 U/ml penicillin G, 100 μg/ml streptomycin sulfate, and 2 mM l-glutamine.

**Construction of the inducible expression vector, preparation of lentiviral vectors, and selection of stable expression clones**

 We used the Tet-On advanced lentiviral vector system (Clontech) and the Tet-Off advanced lentiviral vector system (Clontech) for inducible gene expression. Tcf7l1 and Tcf7l1dN (N-ter 73 amino acid deletion) genes were amplified by PCR from *Homo sapiens* transcription factor 7-like 1 cDNA clone (OriGene Technologies) using Pfx DNA Polymerase (Invitrogen). Tcf7l1-VP16 was prepared by fusing aa 314–471 of Tcf7l1 to the VP16 activation domain. Tcf7l1-En was prepared by fusing aa 314–471 of Tcf7l1 to the repressor domain of Engrailed 1.

Additional materials and methods are presented in Additional file 1: Supplemental information.

## Results

**Fig. 1:**
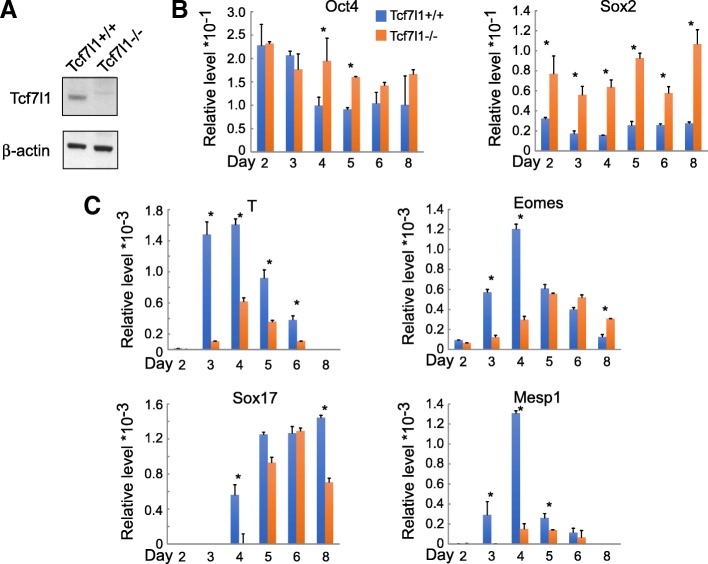
Genetic ablation of Tcf7l1 leads to delayed and partially blocked mesendoderm formation. **a** Western blot confirmation of Tcf7l1 absence in *Tcf7l1*^−/−^ ESCs. **b** Downregulation of *Oct4* and *Sox2* during differentiation is impaired in *Tcf7l1*^−/−^ ESCs. **c** Expression of mesendoderm genes, *T*, *Eomes*, *Sox17*, and *Mesp1*, is delayed and partially reduced in *Tcf7l1*^−/−^ ESCs. Gene expression assayed by real-time RT-PCR. *N* ≥ 3; *p < 0.05 versus control cells

The *Tcf7l1*^−/−^ ESC has a 64-bp deletion in exon 2 of the *Tcf7l1* gene, causing a frameshift and an early termination in translation. Thus, the * Tcf7l1*^−/−^ cell does not express detectable Tcf7l1 (Fig. 1a) [9]. Compensatory upregulation was detected for Tcf7 and Tcf7l2, but not for Lef1 (Additional file 2: Figure S1A). We compared the differentiation course of wildtype *Tcf7l1*^+/+^ and * Tcf7l1*^−/−^ ESCs using the standard embryoid body (EB) culture protocol. Pluripotent genes *Oct4* and *Sox2* decreased during the course of differentiation in *Tcf7l1*^+/+^ cells, but were maintained in *Tcf7l1*^−/−^ ESCs. At later time points, the expression of *Oct4* and *Sox2* was significantly higher in *Tcf7l1*^−/−^ cells (Fig. 1b), suggesting that deletion of Tcf7l1 causes a delay in exiting the pluripotent state. To determine the effects of Tcf7l1 ablation on lineage commitment, we examined the expression of mesoderm and endoderm markers. *Brachyury/T* was significantly lower at all time points, while *Eomes*, *Sox17*, and *Mesp1* showed delayed and lower expression levels in *Tcf7l1*^−/−^ ESCs (Fig. 1c). These data suggest that Tcf7l1 ablation causes a significant delay and incomplete blockage in ESC differentiation.

**Fig. 2:**
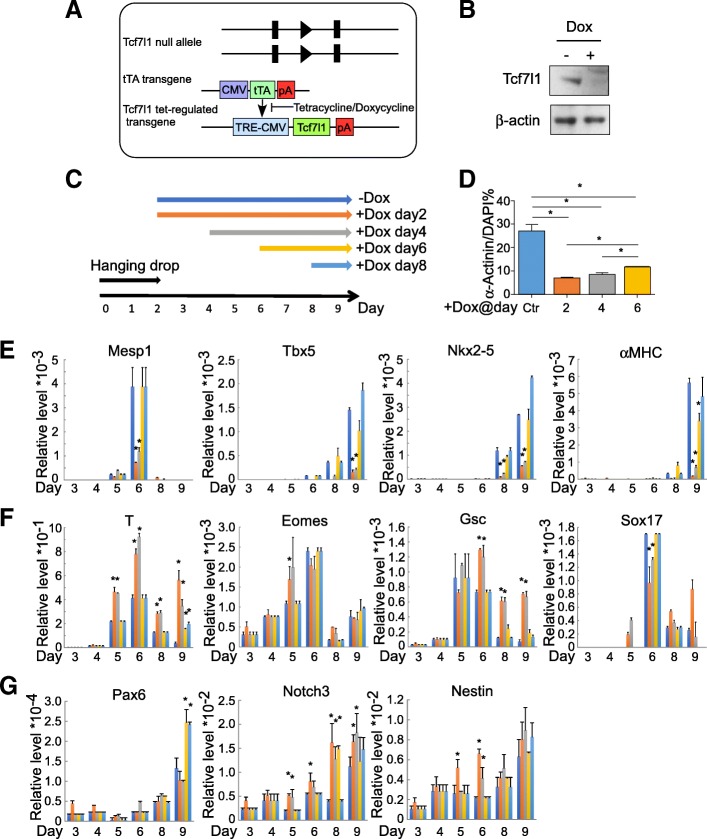
Temporally controlled ablation of Tcf7l1 impaired cardiomyocyte formation without affecting germ layer differentiation. **a** Genetic elements in Tcf7l1-tetoff ESCs. Two transgenes introduced into *Tcf7l1*^−/−^ ESCs: tetracycline-controlled transactivator (tTA) driven by CMV promoter and Tcf7l1 driven by tetracycline responsive promoter (TRE-CMV). Presence of tetracycline or doxycycline blocks activity of tTA transactivator, hence silencing Tcf7l1 transgene. **b** Western blot confirmation of Tcf7l1 transgene silencing by supplemental dox. **c** Scheme of dox supplementation. **d** Ablation of *Tcf7l1* at days 2 and 4 impaired cardiomyocyte formation, evidenced by reduced positive staining for α-Actinin. **e** Cardiac mesoderm marker *Mesp1*, cardiac transcription factors *Tbx5* and *Nkx2–5*, and cardiac structural gene *αMHC* reduced by Tcf7l1 ablation at days 2 and 4, but not ablation at days 6 and 8. **f** Nascent mesendoderm genes *T*, *Eomes*, and *Gsc* elevated, whereas *Sox17 *reduced upon Tcf7l1 ablation at days 2 and 4. **g** Ectoderm genes *Pax6,* *Notch3*, and *Nestin* showed increased expression upon Tcf7l1 ablation at several time points. Gene expression assayed by real-time RT-PCR. *N* ≥ 3; *p < 0.05 versus control cells. CMV cytomegalovirus, Ctr control, DAPI 4′,6-diamidino-2-phenylindole

To determine whether Tcf7l1 is also required for cardiomyocyte formation, in addition to its essential role in transition from pluripotency to differentiation, we engineered a novel ESC model in which Tcf7l1 expression can be ablated in a temporally controlled fashion: into *Tcf7l1*^−/−^ ESCs, we introduced a tetracycline response element (TRE)-controlled Tcf7l1 transgene along with a tetracycline-controlled transactivator (tTA) transgene (Fig. 2a). In this model, supplemental doxycycline (dox) silences Tcf7l1 transgene expression, hence achieving “knock out” (Tcf7l1-tetoff) (Fig. 2b, Additional file 2: Figure S1B). Without dox, the transgene allowed differentiation into cardiomyocytes, evidenced by expression of the cardiac mesoderm marker *Mesp1* at day 6 and of cardiomyocyte genes *Tbx5*, *Nkx2–5*, and *αMHC*at days 8 and 9. Next, we compared the differentiation outcome of Tcf7l1 ablation since days 2, 4, 6, and 8 (Fig. 2c–e). Tcf7l1 ablation since day 2 or 4 significantly reduced the expression of *Mesp1*, *Tbx5*, *Nkx2–5*, and *αMHC*, whereas ablation since day 6 only significantly reduced *αMHC*, suggesting the expression of these genes is dependent on Tcf7l1 (Fig. 2e). Consistently, Tcf7l1 ablation since day 2 or 4 reduced cardiac α-Actinin-positive cardiomyocyte formation (Fig. 2d). To determine whether the impaired cardiomyocyte formation is secondary to defects in mesoderm and endoderm development, we tested the expression of *T*, *Eomes*, *Gsc*, and *Sox17*. Tcf7l1 ablation since day 2 or 4 increased the expression of *T*, *Eomes*, and *Gsc*, supporting that mesoderm and endoderm development are largely intact (Fig. 2f). The increased levels may be secondary to blocked downstream differentiation. In contrast, *Sox17* was downregulated upon Tcf7l1 ablation, consistent with our previous finding that * Sox17* relays cardiogenic signals in the endoderm. We found no changes in early neural markers (*Notch3*, * Pax6*, and *Nestin*) (Fig. 2g), the smooth muscle/myofibroblast marker *ACTA2*, or the panendothelial marker *PECAM-1* upon Tcf7l1 ablation (data not shown), indicating that its obligatory role in cardiomyocyte formation is lineage specific.

Next, we addressed whether the transcription repressor or activator role of Tcf7l1 is involved in activating the cardiomyocyte program. Into *Tcf7l1*^−/−^ ESCs, we introduced three versions of Tcf7l1 transgene: wildtype; Tcf7l1-VP16, a fusion between the Tcf7l1 DNA-binding domain and the VP16 transactivation domain; and Tcf7l1-En, a fusion between the Tcf7l1 DNA-binding domain and the Engrailed repression domain (Additional file 3: Figure S2A). The differentiation timing of the ESCs receiving these transgenes varied, but it was consistent that ESCs expressing Tcf7l1-VP16 showed significantly increased mesodermal markers (*T* and * Mesp1*) compared to ESCs expressing Tcf7l1-En (Additional file 3: Figure S2B). Slightly increased expression of *Nkx2–5* but little effect on *Sox17* was also present. Earlier work in our laboratory [14] established that TCF/LEF proteins cooperate with Oct4 to drive the transcription of *Mesp1*. The significant upregulation of *Mesp1* by Tcf7l1-VP16 suggests that Tcf7l1 may be the responsible TCF/LEF protein.

**Fig. 3:**
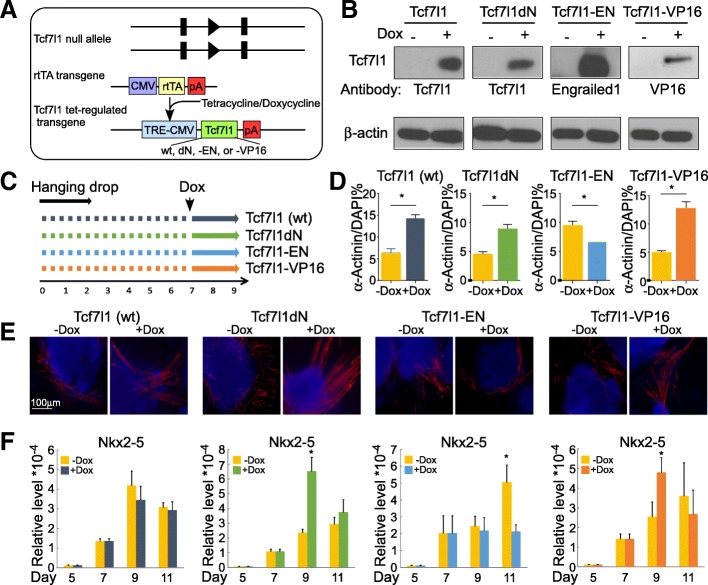
β-Catenin-independent transactivator activity of Tcf7l1 contributes to cardiomyocyte programming. **a** Genetic elements in Tcf7l1-teton ESCs. Endogenous Tcf7l1 alleles are null. Reverse tetracycline-controlled transactivator (rtTA) transgene driven by CMV promoter. Tcf7l1 transgene driven by tetracycline responsive promoter TRE-CMV. In presence of tetracycline/doxycycline, Tcf7l1 transgene is transactivated. Four versions of Tcf7l1 (wt, Tcf7l1dN, Tcf7l1-En, and Tcf7l1-VP16) transgenes compared. **b** Western blot confirmation of transgene induction by 24-h supplemental dox. **c** Scheme of dox supplementation. **d** Tcf7l1dN and Tcf7l1-VP16 augmented formation of α-Actinin-positive cardiomyocytes. **e** Representative α-Actinin staining results of (**c**). **f** Tcf7l1dN and Tcf7l1-VP16 upregulated *Nkx2–5* expression, whereas Tcf7l1-En downregulated it. * Nkx2–5* gene expression assayed by real-time RT-PCR. *N* ≥ 3; *p < 0.05 versus control cells. CMV cytomegalovirus, DAPI 4′,6-diamidino-2-phenylindole, wt wildtype

To further address whether Tcf7l1 is sufficient in triggering the cardiomyocyte differentiation program, we engineered additional ESC models in which Tcf7l1 expression can be activated in a temporally controlled fashion. Into *Tcf7l1*^−/−^ ESCs, we introduced a TRE-controlled Tcf7l1 transgene along with a reverse tetracycline-controlled transactivator (rtTA) transgene (Fig. 3a). The Tcf7l1 transgene is only expressed upon dox supplement (teton). To gain mechanistic insights into the effect of Tcf71, we compared four versions of Tcf7l1: wildtype (wt), mutant with an N-ter deletion abolishing its interaction with β-catenin (Tcf7l1dN), Tcf7l1-VP16, and Tcf7l1-En. Dox supplement for 24 h activated protein expression of the four versions of *Tcf7l1* transgene, with undetectable background (Fig. 3b).

We chose to activate ectopic Tcf7l1 expression at day 7, when *Tcf7l1*^−/−^ cells have passed the stage of mesoderm formation (Fig. 3c–f). This allowed us to evaluate the effect of Tcf7l1 transgenes on cardiomyocyte differentiation. By assaying the cardiac gene *Nkx2–5*, only Tcf7l1dN and Tcf7l1-VP16 activated the cardiomyocyte program (Fig. 3f). Tcf7l1-En downregulated *Nkx2–5*. In immunostaining of α-Actinin, Tcf7l1dN and Tcf7l1-VP16 boosted formation of sarcomeric structures but not Tcf7l1-En (Fig. 3d, e). Ectopic wildtype Tcf7l1 did not activate *Nkx2–5* expression, consistent with the notion that Tcf7l1 is a weaker transactivator compared to Tcf7 and Lef1 [15, 16]. Both Tcf7l1dN and Tcf7l1-VP16 showed more pronounced effects, perhaps because these variants have acquired higher transactivating capacity. These data support our hypothesis that transactivating activity of Tcf7l1 directly contributes to cardiac linage development. Moreover, the de-novo function of Tcf7l1 does not require its interaction with β-catenin in cardiomyocyte differentiation.

**Fig. 4:**
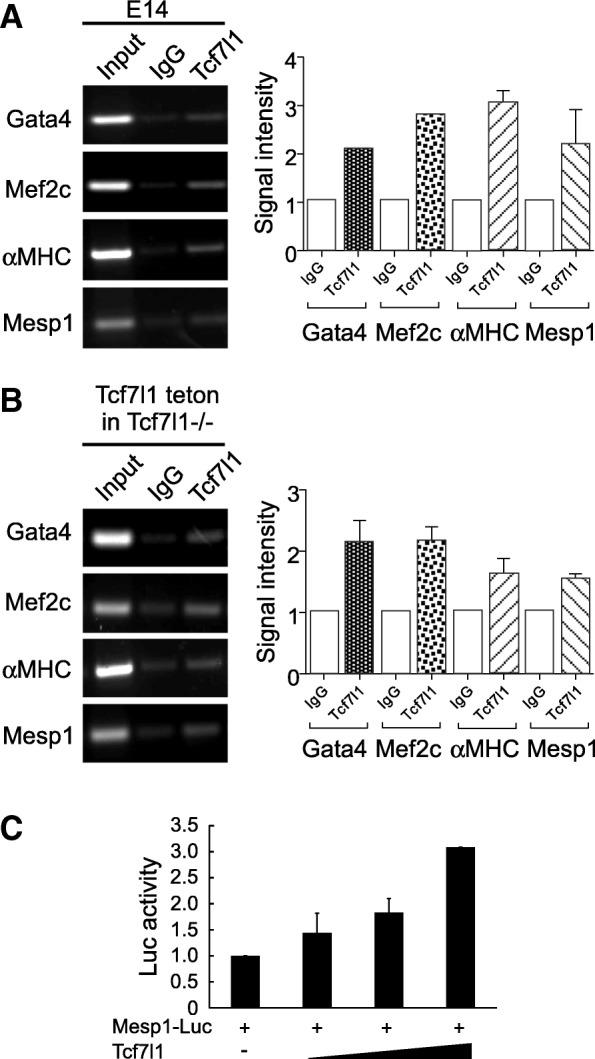
Tcf7l1 interacts with regulatory regions in core cardiac genes. **a** Endogenous Tcf7l1 bound to regulatory regions in *Mesp1*, *Gata4*, *Mef2c*, and *αMHC*. Right: quantification plots. **b** Ectopic Tcf7l1 bound to regulatory regions in *Mesp1*, *Gata4*, *Mef2c*, and *αMHC*. Right: quantification plots. **c** Tcf7l1 dose-dependently activated Mesp1 promoter-driven luciferase reporter

Finally, we tested whether Tcf7l1 directly transactivates important lineage-determining genes in the cardiomyocyte differentiation program. Based on results from a previous whole-genome survey of Tcf7l1-binding sites [17, 18], we selected a number of cardiac genes for chromatin immunoprecipitation PCR (ChIP-PCR) confirmation. Endogenous Tcf7l1 ChIP-PCR revealed enrichment in *Mesp1*, *Gata4*, *Mef2C*, as well as *αMHC*, which are core cardiac transcription factor and structural genes (Fig. 4a). Pulling-down ectopic Tcf7l1 in *Tcf7l1*^−/−^ ESCs also enriched *Mesp1*, *Gata4*, *Mef2C*, and *αMHC* genes (Fig. 4b). Tcf7l1 stimulated the Mesp1-Luc reporter in a dose-dependent manner, supporting that Tcf7l1 functions as a Mesp1 transactivator (Fig. 4c). These data suggest that Tcf7l1 directly binds Mesp1 and other important cardiac transcription factors in driving cardiomyocyte differentiation.

## Discussion

The development of the cardiovascular system requires precisely regulated canonical WNT signaling [19, 20]. As an important downstream factor of WNT, Tcf7l1 is critical in maintaining pluripotency as well as preparing ESCs for gastrulation [21, 22]. However, the function of Tcf7l1 in cardiomyocyte differentiation was unknown, mainly because KO of Tcf7l1 impairs prerequisite steps. In this study, we demonstrate that Tcf7l1 is intrinsically required for the establishment of the cardiomyocyte linage. Our data support that Tcf7l1 contributes to cardiac lineage development as a β-catenin-independent transactivator for Mesp1 and other cardiac lineage-determining genes.

The TCF/LEF family members play important but distinctive roles in embryonic development. Tcf7 is essential for thymocyte differentiation. Homologous deletion of Lef1 led to missing teeth, mammary glands, whiskers, and hair. Tcf7l2 is obligatory for formation of epithelial stem cells in the small intestine. The role and underlying mechanisms of Tcf7l1 are stage dependent and very enigmatic. Tcf7l1 is important for pluripotency maintenance, mesoderm induction, and further specification. It may be specifically required for heart formation: mildly affected *Tcf7l1* null mutants had enlarged hearts, while severely affected mice fail to develop the heart. Our work provides a first-degree approximation of how Tcf7l1 may affect cardiomyocyte formation. The ESC models established in this study may be useful in conditionally manipulating Tcf7l1 expression at the organism level. It was previously reported that Tcf7l1 restricts cardiomyocytes while promoting endothelial specification in zebrafish [23]. Although Tcf7l1 may play different roles in these two species, it is more likely that the loss of Tcf7l1 has triggered compensation by other TCF/LEF factors and the phenotypes reflect varied overall effects. To this end, Moreira et al. [24] demonstrated that a single TCF/LEF factor is sufficient for trilineage differentiation in ESCs, but how the stoichiometry of TCF/LEF factors contributes to cell fate specification and organogenesis warrants additional investigation.

Previous studies have found that Tcf7l1 protein mostly act as a transcriptional repressor, in the absence of β-catenin [9, 22, 25–28]. β-catenin binding releases the repression activity of Tcf7l1, thus maintaining pluripotent cell renewal and triggering gastrulation. However, β-catenin binding seems unessential for gastrulation, as knockin Tcf7l1∆N mutant mice gastrulate normally [3]. In this study, Tcf7l1 worked as a β-catenin-independent transactivator because only Tcf7l1dN and Tcf7l1-VP16 rescued *Tcf7l1*^−/−^ cells for cardiomyocyte differentiation. Although current literature leans heavily toward repressor activity of Tcf7l1, emerging evidence supports that it can also function as a transactivator. It induces LCN2 expression in a β-catenin-independent fashion and drives skin carcinogenesis [29]. Whether the transactivator function of Tcf7l1 requires other cofactors remains unknown.

Both opposite and compensatory effects among the TCF/LEF family members exist in developmental processes, but we were unable to address such effects in this study. Further study is needed to investigate the function of other individual TCF/LEF members, as well as the mechanism of their balanced relationships during cardiomyocyte differentiation.

## Additional files

Additional file 1: Supplemental information. (PDF 179 kb)

Additional file 2: **Figure S1.** (**A**) Expression of Tcf7, Lef1, and Tcf7l2 in *Tcf7l1*^−/−^ ESCs. (**B**) Comparison of conditional transgene expression levels to those in wildtype ESCs. In both Tet-On and Tet-Off systems, expression of transgene is within a comparable range to those in wildtype ESCs (PDF 1121 kb)

Additional file 3: **Figure S2.** Constitutive transactivator activity of Tcf7l1 augmented mesoderm markers. (**A**) Western blot confirmation of ectopic Tcf7l1 expression. (**B**) Differential effects of Tcf7l1-VP16 and Tcf7l1-En on expression of mesendoderm genes, *T* and *Mesp1*, and cardiac transcription factor *Nkx2–5*. Gene expression assayed by real-time RT-PCR. *N* ≥ 3; *p < 0.05 versus control cells (PDF 364 kb)
