# Supplementary material for: Tcf7l1 directly regulates cardiomyocyte differentiation in embryonic stem cells
Source: Stem Cell Res Ther. 2018 Oct 11;9:267. doi: 10.1186/s13287-018-1015-x (PMC6190650; doi:10.1186/s13287-018-1015-x)
Supplement: Supplementary file 1 — Supplemental information. (PDF 179 kb) [file 13287_2018_1015_MOESM1_ESM.pdf]

Supplemental Information

Materials and Methods

Cell Culture

Tcf7l1+/+ and Tcf7l1-/- ESC lines were provided by Dr. Bradley J. Merrill (University of Illinois at Chicago). ESCs were propagated in 0.1% gelatin-coated dishes and cultured with feeder-free ESC medium [DMEM (Gibco) supplemented with 15% FBS (Atlanta Biologicals), 100 U/ml penicillin G, 100 μg/ml streptomycin sulfate, 2 mM L-glutamine, 0.1 mM β-mercaptoethanol, 1× 10^3 U/ml murine leukemia inhibitory factor (LIF, Global Stem)]. Medium was changed daily. To induce EB formation and differentiation, the ESCs were grown as 20 μl hanging droplets (2 × 10^4 cells/ml) in SFDM without LIF [1]. EBs were collected as indicated and the medium was replaced every two days.

293FT cells were cultured in DMEM (Gibco) supplemented with 20% FBS (Atlanta Biologicals), 100 U/ml penicillin G, 100 μg/ml streptomycin sulfate, and 2 mM L-glutamine.

Construction of the Inducible Expression Vector, Preparation of Lentiviral Vectors, and Selection of Stable Expression Clones

We used Tet-On advanced lentiviral vector system (Clontech) and Tet-Off advanced lentiviral vector system (Clontech) for inducible gene expression. Tcf7l1 and Tcf7l1dN (N-ter 73 amino acid deletion) genes were amplified by PCR from Homo sapiens transcription factor 7-like 1 cDNA clone (OriGene Technologies) using Pfx DNA Polymerase (Invitrogen). Tcf7l1-VP16 was prepared by fusing aa 314-471 of Tcf7l1 to the VP16 activation domain. Tcf7l1-En was prepared by fusing aa 314-471 of Tcf7l1 to the repressor domain of Engrailed 1.

To prepare lentiviral infectious particles, the plasmids were cotransfected with packaging plasmid psPAX2 and envelope plasmid pMD2.G at a ratio of 3:2:1 into 293FT cells using FuGENE HD transfection reagent (Promega). The culture supernatant was collected 48h and 72h after transfection. Tcf7l1-/- cells were first infected by pLVX-Tet-On Advanced lentivirus or pLVX-Tet-Off Advanced lentivirus and were selected for G418 (200 μg/ml) resistance. The resulted G418-resistant cells were next infected with pLVX-Tcf7l1, pLVX-Tcf7l1dN, pLVX-Tcf7l1-EN or pLVX-Tcf7l1-Vp16 lentiviruses and were selected with puromycin (1 μg/ml) to generate stable cell lines.

Realtime quantitative RT-PCR

Total RNA was extracted using TRIzol reagent (Invitrogen). Total RNA (100 ng) was subjected to quantitative RT-PCR using Taqman One-Step RT-PCR Master Mix reagent (Eurogentec) and 7900 HT Fast-Real-Time PCR System (Applied Biosystems). Copy number for each transcript is calculated relative to that of GAPDH. Sequences of primers and probes are as the following:

Gene	Forward primer	Probe	Reverse primer
Oct4	CACGAGTGGAAAGCAACTCAGA	CTCTGAGCCCTGTGCCGACCG	TCTCCAACTTCACGGCATTG

Sox2	TGGACAGCTACGCGCACA	CTGCCGTTGCTCCAGCCGTTC	GCTGCTCCTGCATCATGCT
Eomes	GCCGTCTGCGATTCGCT	AGCATGCAGTTGGGAGAGCA	GGGCAGGTTCACCGAGCT
T	AACCACCGGTCATCGCC	TACCCCAGCCCCTATGCTCATCGG	TCCGCATAGGTTGGAGAGCT
Gsc	CTGGCCAGGAAGGTGCAC	TTCGGGAGGAGAAGGTGGAGGTCTG	CTTGGCTCGGCGGTTCTTA
Mesp1	GGCACCTTCGGAGGGAGTAG	TCCTGGAAGAGGCGGCAGTGATACC	CCCGGGATGCCATGT
Sox17	TCGGTCTGGAGAGCCATGAG	TACGCCAGTGACGACCAGAGCCAGC	CCACCACCTCGCCTTTCAC
Notch3	CTGGGAATGAAGAACATG	CATTCAAGTCTGTGACCACCTCC	CTACCTTCAGTCTCTTGG
Pax6	GACTGCCAGCTTCCATCCAC	CCTCGCCTCCAGCCTCAGCCG	AACACACCAACTTTCGCAAGA TAG
Nestin	GCTTGAAAGACTGGTAGAGA AAGAG	CTCCAGAAGAAGACCAGCAGGCGTT	CACCTGGTCCTCTGCTTCTTC
Tbx5	CAGGCTGCCTTCACCCAG	AGGGCATGGAAGGAATCAAGGTGTTT CT	CAGCCACAGTTCACGTTCATG
Nkx2-5	CCTCGGGCGGATAAAAAAGA	CGCGCTGCAGAAGGCAGTGG	GCCATCCGTCTCGGCTT
αMHC	GAATGACGGACGCCCAGATG	TTGTCATCAGGCACGAAGCACTCCG	ACGACCTTGGCCTTAACATAC TC
Gapdh	ACTGGCATGGCCTTCCG	TTCCTACCCCCAATGTGTCCGTCGT	CAGGCGGCACGTCAGATC

Western Blots

Cells were lysed in RIPA buffer [50 mM Tris-HCl (pH 8.0), 150 mM NaCl, 1% NP-40, 0.5% sodium deoxycholate, 0.1% SDS], and the proteins were separated on a 4-12% Bis-Tris gel (Life Technologies) and transferred onto nitrocellulose membranes. The membranes were incubated with the following primary antibodies at 4°C overnight: goat monoclonal antibody to Tcf7l1 (1:2,000; Santa Cruz Biotech sc-8635), rabbit monoclonal antibody to engrailed (1:1,000; Santa Cruz Biotech sc-28640), mouse monoclonal antibody to VP16 (1:1,000; Santa Cruz Biotech sc-7545) and goat monoclonal antibody to HRP-conjugated β-actin (1:2,000; Santa Cruz Biotech). The membranes were next incubated with horseradish peroxidase-conjugated secondary antibodies to the appropriate IgG (1:2,000; Life Technologies) for 90 min. Bound antibodies were visualized with enhanced chemiluminescence reagents (Amersham Biosciences).

Immunostaining

Cells were fixed with 4% paraformaldehyde for 10 min, followed by 0.1% Triton X-100 in PBS for 30 min at room temperature. The samples were then blocked with 10% normal goat serum in 0.1% Triton X-100/PBS for 30 min at 37°C. Primary mouse monoclonal antibody to α-Actinin (1:100; Novus Biologicals) were incubated at 4°C overnight. Secondary antibody Alexa Fluor 594 goat anti-mouse IgG (H+L) antibody (1:500; Life Technologies) was added and incubated at room temperature for 1h.   The samples were next incubated with DAPI for 5 min. Images were captured with an Nikon Fluorescence microscope.

Luciferase Reporter Assay

Construction of Mesp1-Luc was previously described [2]. Different doses of pCMV6-XL5-Tcf7l1 (0.5 μg, 1 μg and 2 μg), 1μg Mesp1-Luc reporter, and 0.05 μg internal control plasmid pRL-CMV were transfected into 293FT cells using FuGENE HD transfection reagent (Promega). The cells were lysed in passive lysis buffer (Promega) 24h post-transfection. The assay was performed using the Dual-Luciferase Reporter Assay System (Promega). The luciferase activity was read in a Turner Biosystems 20/20 Luminometer.

Chromatin Immunoprecipitation (ChIP) Assay

E14 ESCs and Tcf7l1 tet-on (in Tcf7l1-/-) ESCs went differentiated for 8 days. For Tcf7l1 teton ESCs, doxycycline was added on day 0 of differentiation. Differentiated cells were crosslinked with 1% formaldehyde for 15 min at room temperature and this cross-linking reaction was inactivated by the addition of glycine to a final concentration of 125 mM, which was followed by a 10 mins incubation at room temperature. Cells were washed twice with ice-cold PBS and harvested using a scraper. The cells were next centrifuged at 3500 rpm for 5 mins at 4°C, and the pellet was snap-frozen in liquid nitrogen and stored at -80°C until the day of use. Cell pellets were resuspended in cell lysis buffer containing protease inhibitors (5 mM PIPES pH 8.0, 85 mM KCl, 0.5% NP-40), incubate on ice for 15 mins and centrifuged at 4°C for 5 mins at 5000 rpm. The cell pellet was resuspended in nuclear lysis buffer containing protease inhibitors (50 mM Tris pH 8.0, 10 mM EDTA, 1% SDS), and incubated for 15 mins on ice. Sonication was performed using Diagenode Bioruptor Plus for 10 times on 30-second on and 30-second off pulses. Samples were kept on ice at all times. The cell lysate was centrifuged at 14000 rpm for 15 mins at 4 °C and the supernatant transferred to a fresh tube. The supernatant was diluted in lysis buffer (150 mM NaCl, 25 mM Tris pH8.0, 5 mM EDTA, 1% Triton-X 100, 0.5% Deoxycholate) to bring down the amount of SDS to 0.125%. The diluted lysate was precleared with IgG for 1 h and then Protein A agarose for another 1 h at 4 °C. After a brief centrifugation, the supernatant was transferred to a fresh tube and incubated with the primary antibody overnight at 4 °C. 40 μL protein A agarose bead was added and incubated for another 1 h at 4 °C on the next day. After several washes: 3 times with RIPA buffer (150 mM NaCl, 50 mM Tris pH 8.0, 1 mM EDTA, 1% NP40, 0.1% SDS, 0.5% Deoxycholate), 1 time with high salt buffer (500 mM NaCl, 50 mM Tris pH 8.0, 1 mM EDTA, 1% NP40, 0.1% SDS, 0.5% Deoxycholate), 1 time with LiCl-wash buffer (250 mM LiCl, 50 mM Tris pH8.0, 1 mM EDTA, 1% NP40, 0.5% Deoxycholate) and 2 times with TE buffer, bound complexes were eluted by heating at 65 °C with occasional vortex, and crosslinks were reversed by incubation at 65 °C overnight. Eluted protein-DNA complexes were treated with RNase A and Proteinase K sequentially. Following phenol-chloroform extraction and ethanol precipitation, DNA was dissolved in H_2O.

The following PCR primers were used to amplify regulatory regions of indicated cardiac genes: Mesp1 Forward, GGCACCTTCGGAGGGAGTAG, Mesp1 Reverse, CCCGGGATGCCCATGT; Mef2c Forward, TCCACTCCCCCATTGGACT, Mef2c Reverse, TGCGCTTGACTGAAGGACTTT; αMHC Forward, GAATGACGGACGCCCAGATG, αMHC Reverse, ACGACCTTGGCCTTAACATACTC; Gata4 Forward, TTAGCCCAGTTCTTGCGTGT, Gata4 Reverse, CGGACCGGCTTGTCTTTAGT.

1. Liu Y, Chen L, Diaz AD et al. Mesp1 Marked Cardiac Progenitor Cells Repair Infarcted Mouse Hearts. **Scientific reports**. 2016;6:31457.
2. Li Y, Yu W, Cooney AJ et al. Brief report: Oct4 and canonical Wnt signaling regulate the cardiac lineage factor Mesp1 through a Tcf/Lef-Oct4 composite element. **Stem Cells**. 2013;31:1213-1217.
