# Supplementary figures and images for: Tcf7l1 directly regulates cardiomyocyte differentiation in embryonic stem cells
Source: Stem Cell Res Ther. 2018 Oct 11;9:267. doi: 10.1186/s13287-018-1015-x (PMC6190650; doi:10.1186/s13287-018-1015-x)

**A**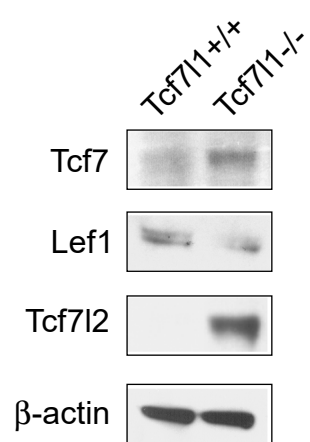**B**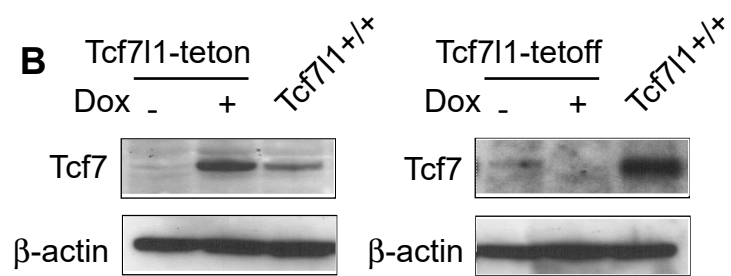

supplemental FigS1.

Supplement: Supplementary file 2 — Figure S1. (A) Expression of Tcf7, Lef1, and Tcf7l2 in Tcf7l1−/− ESCs. (B) Comparison of conditional transgene expression levels to those in wildtype ESCs. In both Tet-On and Tet-Off systems, expression of transgene is within a comparable range to those in wildtype ESCs (PDF 1121 kb) [file 13287_2018_1015_MOESM2_ESM.pdf]

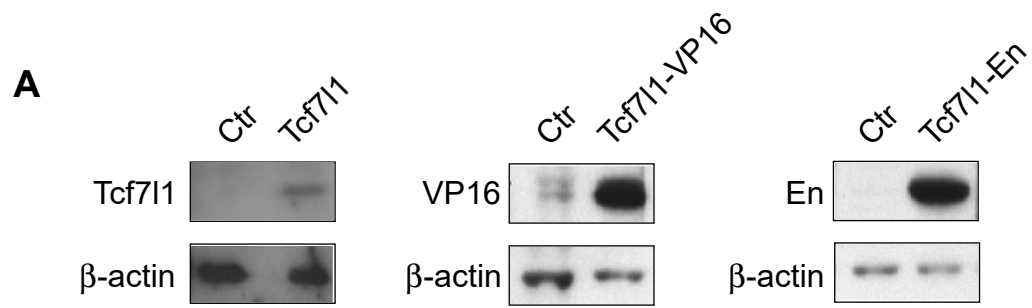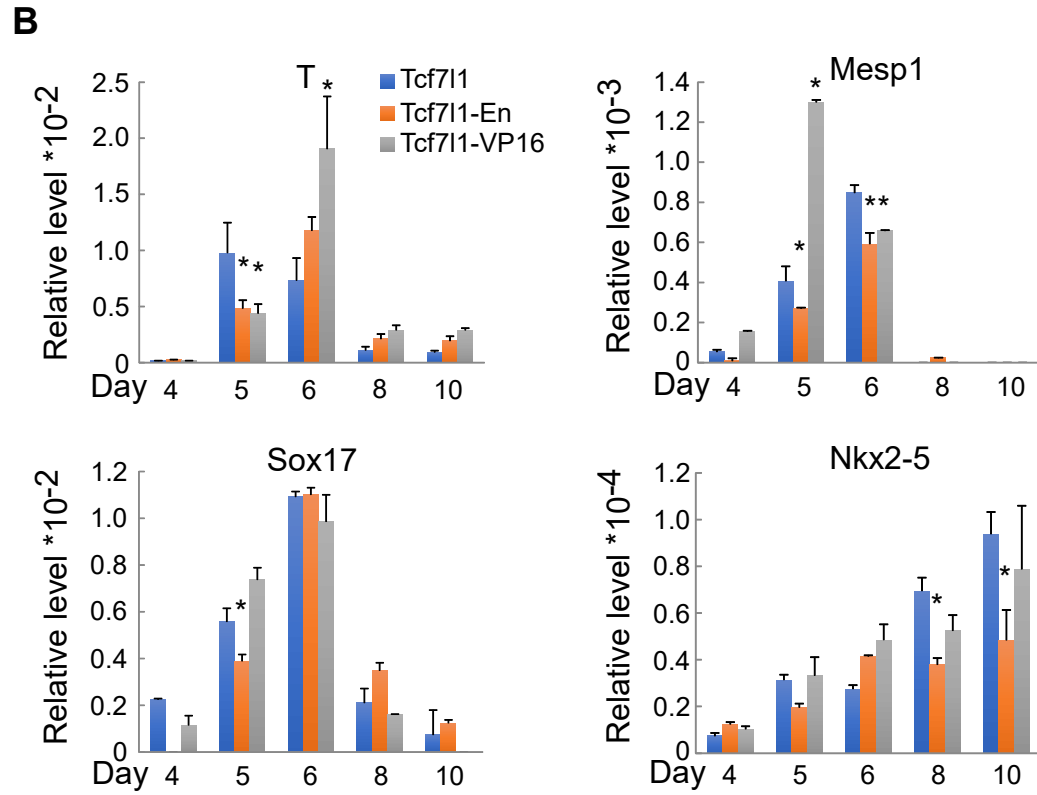

supplemental FigS2.

Supplement: Supplementary file 3 — Figure S2. Constitutive transactivator activity of Tcf7l1 augmented mesoderm markers. (A) Western blot confirmation of ectopic Tcf7l1 expression. (B) Differential effects of Tcf7l1-VP16 and Tcf7l1-En on expression of mesendoderm genes, T and Mesp1, and cardiac transcription factor Nkx2–5. Gene expression assayed by real-time RT-PCR. N ≥ 3; *p < 0.05 versus control cells (PDF 364 kb) [file 13287_2018_1015_MOESM3_ESM.pdf]
